# A Data-Driven Design Evaluation Tool for Handheld Device Soft Keyboards

**DOI:** 10.1371/journal.pone.0107070

**Published:** 2014-09-11

**Authors:** Matthieu B. Trudeau, Elsie M. Sunderland, Devin L. Jindrich, Jack T. Dennerlein

**Affiliations:** 1 University of Calgary, Calgary, Alberta, Canada; 2 Harvard School of Public Health, Boston, Massachusetts, United States of America; 3 Harvard School of Engineering and Applied Sciences, Cambridge, Massachusetts, United States of America; 4 California State University, San Marcos, California, United States of America; 5 Northeastern University, Boston, Massachusetts, United States of America; University of Perugia, Italy

## Abstract

Thumb interaction is a primary technique used to operate small handheld devices such as smartphones. Despite the different techniques involved in operating a handheld device compared to a personal computer, the keyboard layouts for both devices are similar. A handheld device keyboard that considers the physical capabilities of the thumb may improve user experience. We developed and applied a design evaluation tool for different geometries of the QWERTY keyboard using a performance evaluation model. The model utilizes previously collected data on thumb motor performance and posture for different tap locations and thumb movement directions. We calculated a performance index (PI_TOT_, 0 is worst and 2 is best) for 663 designs consisting in different combinations of three variables: the keyboard's radius of curvature (R) (mm), orientation (O) (°), and vertical location on the screen (L). The current standard keyboard performed poorly (PI_TOT_ = 0.28) compared to other designs considered. Keyboard location (L) contributed to the greatest variability in performance out of the three design variables, suggesting that designers should modify this variable first. Performance was greatest for designs in the middle keyboard location. In addition, having a slightly upward curve (R = −20 mm) and orientated perpendicular to the thumb's long axis (O = −20°) improved performance to PI_TOT_ = 1.97. Poorest performances were associated with placement of the keyboard's spacebar in the bottom right corner of the screen (e.g., the worst was for R = 20 mm, O = 40°, L =  Bottom (PI_TOT_ = 0.09)). While this evaluation tool can be used in the design process as an ergonomic reference to promote user motor performance, other design variables such as visual access and usability still remain unexplored.

## Introduction

Owners of small handheld devices such as smartphones are on the rise [1]. These devices are being used for text communication, with 80% of people using their cell phone to send or receive text messages, and 50% to send or receive email [2]. According to an observational study of 859 college students, 44% of handheld users hold the device with a single hand while the thumb of the same hand interacts with the screen [3]. Single-handed use of a mobile device requires a different technique than operating a computer, yet keyboard layouts for both devices are similar, with the keyboard located below and orthogonal to the display. This provides familiarity for the user when accomplishing tasks on their smartphone that they commonly accomplish or used to accomplish on a computer workstation keyboard. With the high prevalence of handheld devices, a keyboard design that is specific to the small form factor should be developed with the goal of improving the ergonomics of the interaction, thus promoting user experience. More specifically, handheld device keyboard design solutions that consider the physical capabilities and motor performance of the thumb must be investigated.

Previous studies in the fields of ergonomics, human factors and biomechanics, have found that thumb performance metrics such as speed and precision vary according to the movements constrained by the phone's design. Performance, as a function of movement time, varies with thumb movement orientation [4]. Similarly, thumb motor performance as calculated using a modification of Fitts' Law varies with movement direction [5]. For right-handed users, performance was best for movements in the top right/bottom left orientation of the phone and worst for the top left/bottom right orientation. Additionally, greater perceived effort and poorer tapping speed are reported for thumb movements along the top left/bottom right orientation for right-handed users [6]. Keys located toward the middle of the phone generally lead to lower transition times, and were also more convenient to press for the user [7]. This result is consistent with thumb reach envelopes on small handheld devices [8]. Trudeau et al. (2012b) further report that the association between key location and thumb motor performance may be explained by the thumb and wrist postures required to reach the keys. Neutral thumb postures were found to lead to greater motor performances than when the thumb was either flexed or extended [9]. These studies provide general guidelines as to where keys should be located, but they do not provide practical keyboard designs or design guidelines that promote the thumb's physical capabilities for more complex tasks such as typing.

The first step in designing a handheld keyboard that promotes thumb use is to identify keyboard design variables that can be modified, and then determine how these modifications affect thumb performance. For example, a few studies have proposed mobile device keyboard layouts that deviate from the standard QWERTY keyboard in an attempt to increase productivity [10]–[12]. However, alternative keyboard layouts that deviate from the standard QWERTY layout pose usability problems because they greatly affect important design considerations such as learnability and familiarity. Design modifications aimed at promoting thumb use can be made without changing the QWERTY key layout, which is the focus of the present study.

The design of keyboard layouts is, and has been, a very popular topic in the ergonomics literature, especially with respect to the computer workstation. Such literature has been influential in modifying the design of the keyboard for improved user experience. Since the tasks accomplished on the computer keyboard and on handheld device keyboards are similar, providing background on the evolution of computer keyboards is useful to fully understand the importance and context of the study presented here. The split keyboard design is an example of a design modification to the computer keyboard that has stemmed from research in workstation ergonomics in the 1960's and 70's. The split keyboard design promotes a neutral wrist posture compared to the conventional keyboard, and has effectively yielded health benefits and became the number one selling keyboard in the US as of 2006 [13]. In a similar way, modifying design variables for the handheld device keyboard may lead to similar benefits. Design variables that can be modified for handheld device QWERTY keyboards include its curvature, orientation and location on the screen. The investigation of how each one of these design variables affects thumb performance can be accomplished without the need for testing on human subjects since data in the literature can be used to develop a design evaluation tool. As a result, many more possible layouts can be evaluated without the time and cost of running human participant research. From these, specific data points can be selected to evaluate in future studies.

Several studies in the human-computer interaction literature have proposed design evaluation tools for keyboard layouts [12], [14]–[17]. These involve models to predict the performance of a user for accomplishing a task based on the estimated time needed to accomplish elementary subtasks. A frequently cited design evaluation tool is the Keystroke-Level Model (KLM) [14], which has been used extensively to evaluate workstations or keyboard layouts based on the predicted total time it would take an expert user to accomplish a task. In calculating the predicted performance of a complex task (e.g., typing), tools such as Fitts' Law [18], or modifications of Fitts' Law [19], [20], can be used to calculate the performance of subtasks (e.g., taps) based on a user's speed and accuracy to reach a target. Additionally, several studies have proposed new key layouts based on key usage frequency and the physical and cognitive requirements for completing a task [12], [17]. Design evaluation tools are popular because they can save substantial time and money for designers wishing to narrow their range of design options to ergonomic solutions before user testing. To our knowledge, no model has yet to evaluate different designs of a QWERTY keyboard layout on a mobile device based on biomechanical data from real users.

Therefore, our main objective was to describe and demonstrate a design evaluation tool that we created based on data from an experiment in which we measured thumb motor performance and posture for different thumb movement directions and tap locations on the screen [9]. We calculated performance indices from a simulated typing task for designs consisting of combinations of 3 design variables: the keyboard's radius of curvature, it's orientation with respect to the screen, and its vertical location on the screen. The tool described here can be used to evaluate how well different handheld device keyboard designs fit the thumb's physical capabilities in terms of predicted performance and posture. Given its resemblance to a computer workstation's layout, we hypothesized that the predicted performance for the standard keyboard design at the base of the handheld device can be improved upon to fit the physical capabilities of the thumb. Moreover, by identifying layouts that maximize predicted performance, the tool could contribute to providing data-driven predictions of usability and ergonomics before the prototype development and usability testing stages. Therefore, the design evaluation tool proposed in this study provides a solution for streamlining the design process.

## Methods

**Figure 1:**
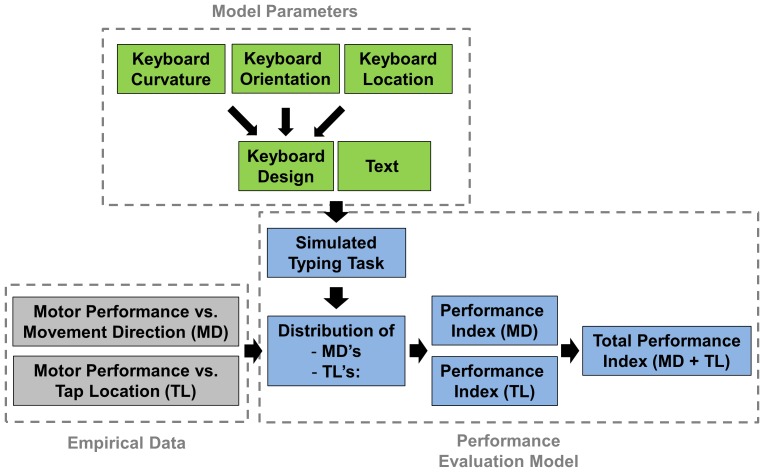
Flow chart describing the design evaluation tool.

We predicted a user's typing performance for different keyboard designs by emulating the various key positions required to type a certain text and using existing thumb motor performance data that were previously collected in experimental protocols [5], [9]. The tool has three basic components: (1) empirical thumb motor performance data, (2) model parameters that include three keyboard design variables and a text, and (3) a performance evaluation model that involves a simulated typing task and calculation of a performance index for a given design (Figure 1). The results from this paper do not directly relate to the performance indices of particular human subjects, but rather they relate to model-predicted performance indices.

### Empirical Data

**Figure 2:**
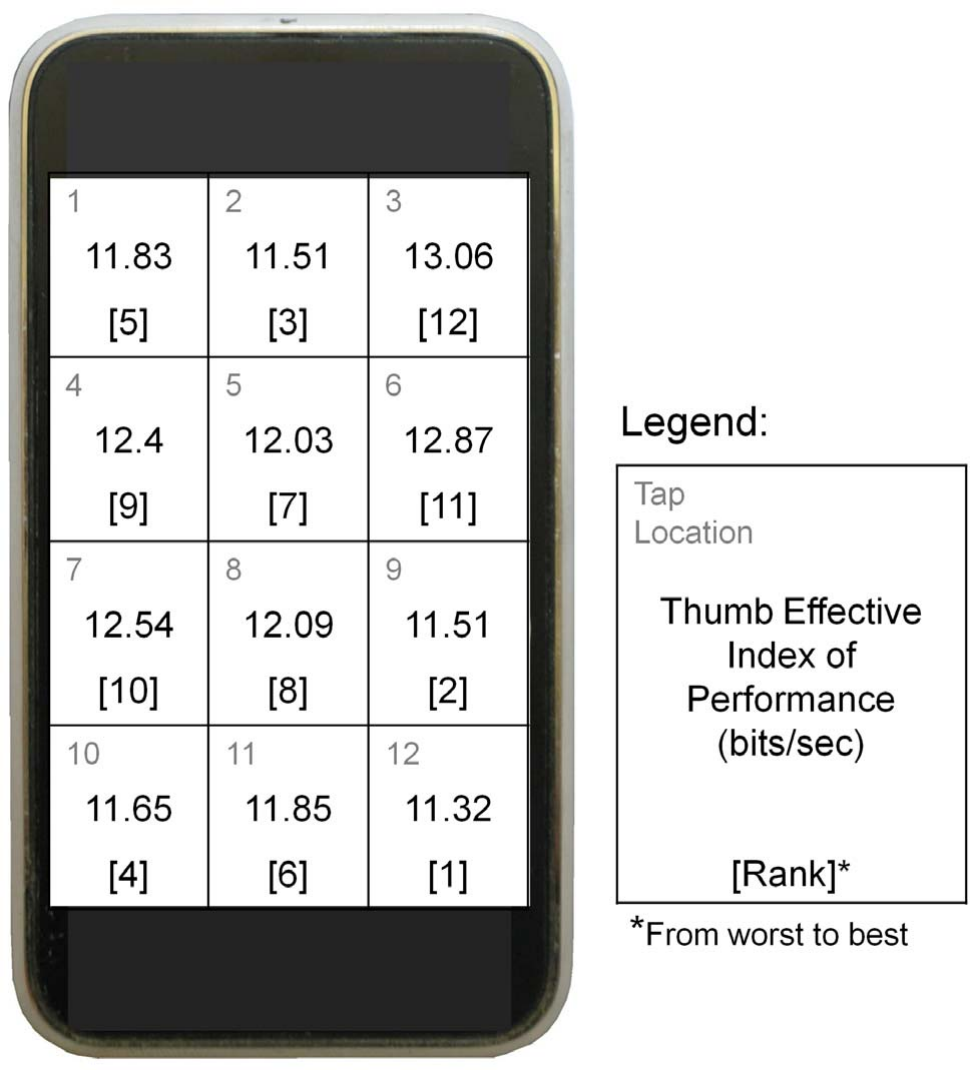
Average values for effective index of thumb performance (bits/sec) and their rank for each tap location determined by Trudeau et al., 2012b, used in the calculation of the tap location performance index (adapted from Trudeau et al., 2012b).

Empirical data from our prior research demonstrated that thumb motor performance varies for different tap locations and thumb movement directions during reciprocal tapping tasks [5], [9]. These data served as the basis of our performance evaluation model. Using a modified version of Fitts' Law [18]–[20] we calculated an effective index of performance based on the speed and accuracy of 10 right-handed participants who accomplished trials that consisted in a reciprocal thumb tapping task between 2 of 12 different emulated keys on an Apple iPhone 3 (Figure 2), and then repeated this task covering a representative sample of the various possible key locations on the screen [9]. The selection and presentation of the key pairs was randomized for every participant in order to achieve a representative sample of all the possible incoming tap directions for each key. An average of 47±6 trials were analyzed per participant. Instructions to participants were to “complete the task as fast and as accurately as possible”. Participants could adjust their grip between trials. For each reciprocal tapping trial, 6 seconds of data collection started once the subject indicated that they were comfortable with the tapping task. The experiment duration was 1 hour 30 minutes, and participants rested for 90 seconds after every 15 trials.

Tapping at the top right corner of the device (tap locations 3 and 6) was associated with the greatest performance (Figure 2), and tapping at the bottom right corner (tap location 12) was associated with the lowest performance. Data from Trudeau et al. (2012b) provide an association between thumb effective index of performance and movement direction for the model we present in this paper (Table 1). The movement direction that led to the best performance was toward the top right corner and the direction that led to the worst performance was toward the bottom right corner of the phone for right handed users.

**Table 1:** Average values for effective index of thumb performance (bits/sec) and their rank for each thumb movement direction, used in the calculation of the movement direction performance index (Trudeau et al., 2012a and Trudeau et al., 2012b).

Thumb Movement	Thumb Effective	Rank
Direction^a^	Index of Performance (bits/sec)	(from worst to best)
N to S	11.17	2
S to N	12.93	7
W to E	11.61	3
E to W	11.84	4
NE to SW	12.50	6
SW to NE	13.22	8
NW to SE	11.13	1
SE to NW	11.93	5

a Movement directions refer to the cardinal directions (i.e., NW: North-West, SE: South-East, etc.).

We previously found an association between thumb motor performance and thumb/wrist postures required to reach different key locations on the screen [9]. Taps requiring extreme thumb postures in flexion and extension were associated with poor effective performances, and taps in which the thumb was in a neutral posture were associated with strong performances. In that paper we suggested that thumb/wrist posture may therefore be one of the factors involved in explaining the associations described above. The methods used to collect the data used in elaborating the evaluation tool defined here are described in more detail in Trudeau et al. (2012b).

### Keyboard layout design metrics

**Figure 3:**
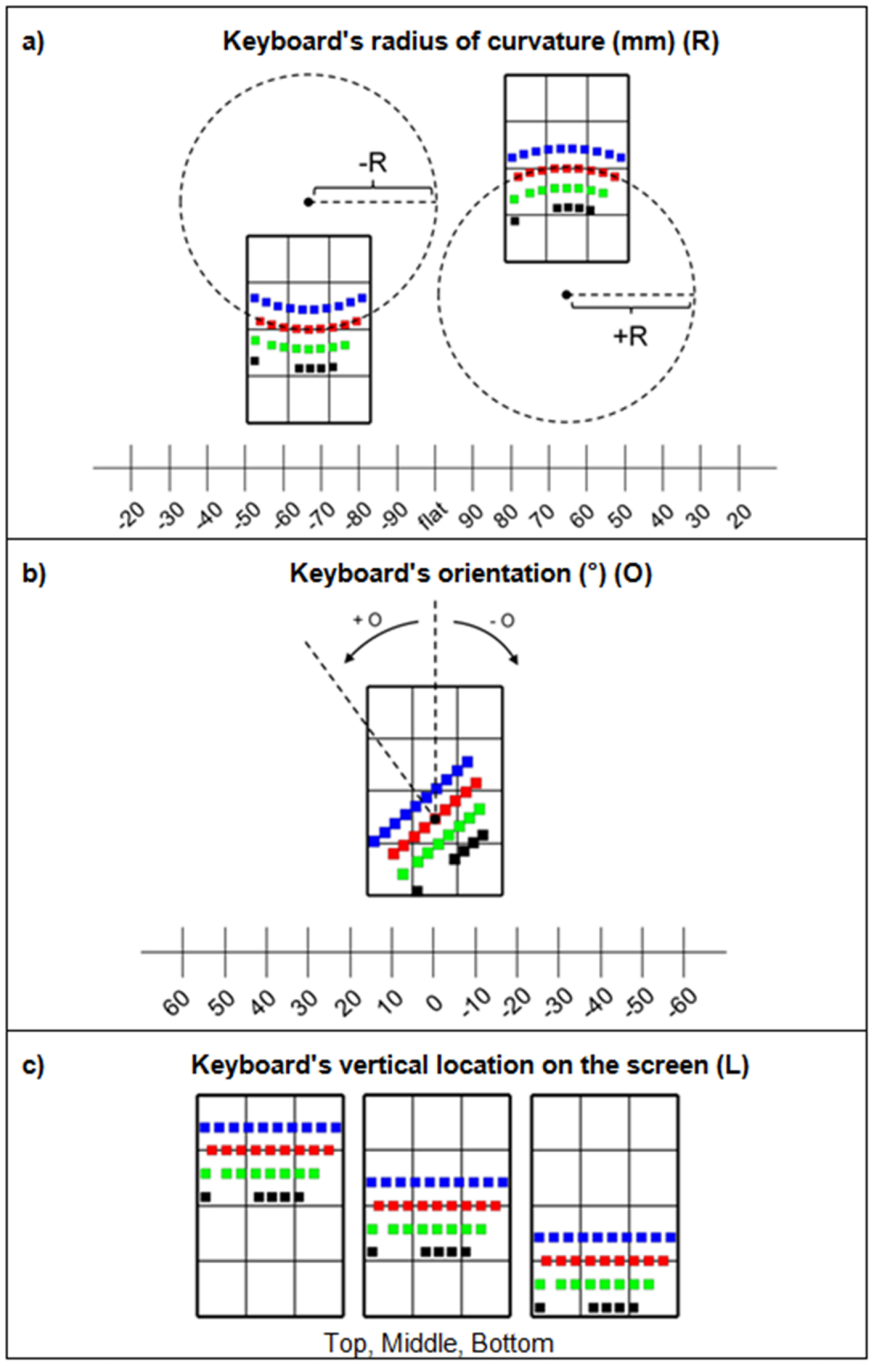
Design parameters: (a) the keyboard's radius of curvature (R) has 17 levels; (b) the keyboard's orientation with respect to horizontal (O) has 13 levels; (c) the keyboard's location on the screen has 3 levels.

**Figure 4:**
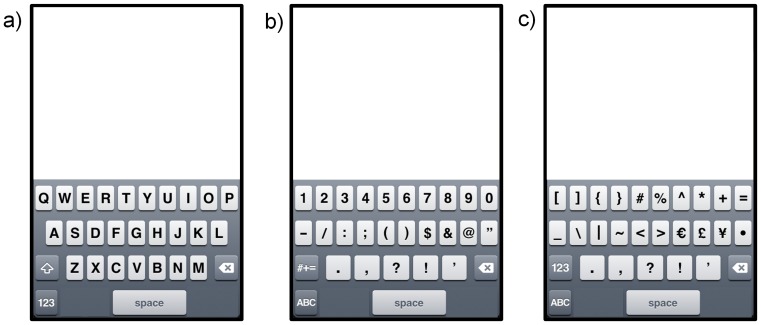
Key layout for Apple iOS 5.1's touch keyboard for the iPhone 4S (Apple Inc.) used in for the simulated typing task. (a) Letters layout; (b) numbers layout; (c) symbols layout.

We examined three fundamental keyboard layout features: (1) radius of curvature (R) (mm), (2) orientation with respect to the device's vertical midline (O) (°), and (3) vertical location on the screen (L) (Figure 3). We started with the standard key layout from Apple Inc.'s iOS 5.1 for the iPhone 4S (Figure 4). We defined keyboard radius of curvature (R) as the circular curvature of the keyboard's home row. The top and bottom rows and the spacebar were curved such as to follow the curvature of the circle's center (Figure 3a). Positive R's corresponded to a keyboard that was curved down, whereas negative R's corresponded to a keyboard that was curved up. Keyboard orientation (O) was about the home row's center key (the “G” key). Counter-clockwise orientations were positive O's, and clockwise orientations were negative O's (Figure 3b). We defined the keyboard's vertical location as the location of the home row's middle key (the “G” key) with respect to the bottom edge of the screen (Figure 3c). Key size was kept constant across layouts. Each keyboard design considered in this study consisted in a combination of a specific keyboard radius of curvature (R), orientation (O), and vertical location (L).

### Performance Evaluation Model

For every keyboard design, each one of the keyboard's letters and symbols was located within the area defined by one of the 12 key locations on the screen's surface area (Figure 2) for which we have previously calculated an effective index of performance (IPe) [9]. Additionally, in order to type a given text, a user would have to move their thumb in one of the 8 movement directions (Table 1) for which we have previously calculated an effective index of performance (IPe). Our assumption here is that these taps can be translated to the non-repetitive motion of typing rather than tapping. Therefore, we assume that the data source can be directly applied to the different keyboard designs presented in this study.

We calculated the distribution of predicted movement directions and tap locations that would arise from a user typing a given text on a given keyboard design. Since we considered a simulated typing task, users did not actually accomplish this typing task. Rather, we predicted typing performance based on previously collected tapping data using a task analysis approach in which a task (i.e., typing a text using the thumb on a phone's keyboard) can be described as a series of smaller subtasks (i.e., tapping onto a key by moving the thumb from one location on the phone to the location of the target key). The simulated typing task involved entering 375 characters of text. This length led to consistent performance index results across different text contents of the same length. Shorter texts led to substantial variability in the results across texts. The text used was an excerpt from the Huffington Post [21], and is included in Appendix S1. We then calculated a performance index (PI_TOT_) for the given design using these distributions and the associations from the empirical data. Note that the acronym “PI” refers to a different variable than the acronym “IPe” in that “PI” refers to a performance index that is calculated using the effective motor performance indices “IPe” that were calculated from Fitts' Law, as described below. Keying punctuation, numbers or symbols involved keying one or two function keys to the left of the spacebar first, entering a capital letter involved keying the “shift” key first, and so on (Figure 4). The model assumed that the “auto-correct”, “auto-capitalization”, and “auto-complete” functions were turned off for the simulated typing task. The space bar was modeled as 4 adjacent keys, and the one considered as being tapped in the simulated typing task was the closest in distance to the key previously tapped.

For the simulated typing task, we categorized each tap into 1 of 12 different key locations (Figure 2) and 1 of 8 different thumb movement directions (Table 2) based on the locations of the previous key (i.e. the previous letter, number or symbol tapped) and the target key (i.e. the next letter, number or symbol tapped). This allowed us to predict a distribution of all the tap locations and movement directions involved in typing the text on the given keyboard design.

**Table 2:** Results from the Kruskal-Wallis tests showing the relative variability of each design variable on the predicted performance: keyboard radius of curvature (R), keyboard orientation with respect to vertical (O), and keyboard vertical location on the screen (L).^a^

Effect	H-statistic	p-value
R	13.01	0.67
O	107.02	**<0.01**
L	299.21	**<0.01**

a Statistically significant effects are in bold.

#### Calculation of the Performance Index

For each keyboard design, we calculated gross performance indices for both tap location (PI_TL,gross_) and movement direction (PI_MD,gross_) by multiplying the distributions (D) calculated from the simulated typing task by the rank (Rk) of the respective effective motor performance (IPe) for every tap location and thumb movement direction (i.e., Rk(IPe_key_) and Rk(IPe_mov_), respectively) (Figure 2 and Table 1). Therefore, locations/directions associated with high motor performances substantially increased the performance index, while locations/directions associated with poor motor performances contributed little to the performance index. We did not consider taps that involved no change in tap location in the calculation of the movement direction performance index. Next, we normalized the gross performance indices by dividing them by the total number of taps (Tt) involved in the typing task:
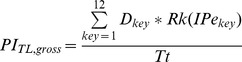





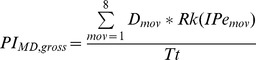



We then ranked the keyboard designs in ascending order according to the calculated tap location and movement direction indices (PI_TL,gross_ and PI_MD,gross_). Next, we normalized each design's rank by the total number of designs considered (i.e., 663). This resulted in a tap location performance index (PI_TL_) and movement direction performance index (PI_MD_) based on a relative scale from 0.00 to 1.00. Finally, we summed PI_TL_ and PI_MD_ to provide a total performance index for each keyboard layout (PI_TOT_), with 0.00 being the poorest possible performance and 2.00 being the best:
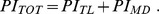



### Design Parameter Space Explored

To demonstrate the capabilities of the evaluation tool, we calculated performance indices (PI_TOT_) for 663 different keyboard designs. We considered 17 levels of the keyboard's radius of curvature (R), 13 levels of the keyboard's orientation (O), and 3 levels of the keyboard's vertical location (L), for a total of (17×13×3) 663 different keyboard designs. We chose these limits for R and O to minimize the effect on the keyboard's usability (e.g., we subjectively assessed that an orientation greater than 60° produces a keyboard that may be unfamiliar for users due to its close-to-vertical orientation.). We selected the bottom, middle, and top keyboard locations such that the home row's middle key (the “G” key) was located one fourth, half-way, and three fourths of the way up from the bottom edge of the phone's screen, respectively (i.e., 19, 38, and 57 mm from the bottom edge of the screen, which is 76 mm long for the iPhone 4S).

### Analysis

**Figure 5:**
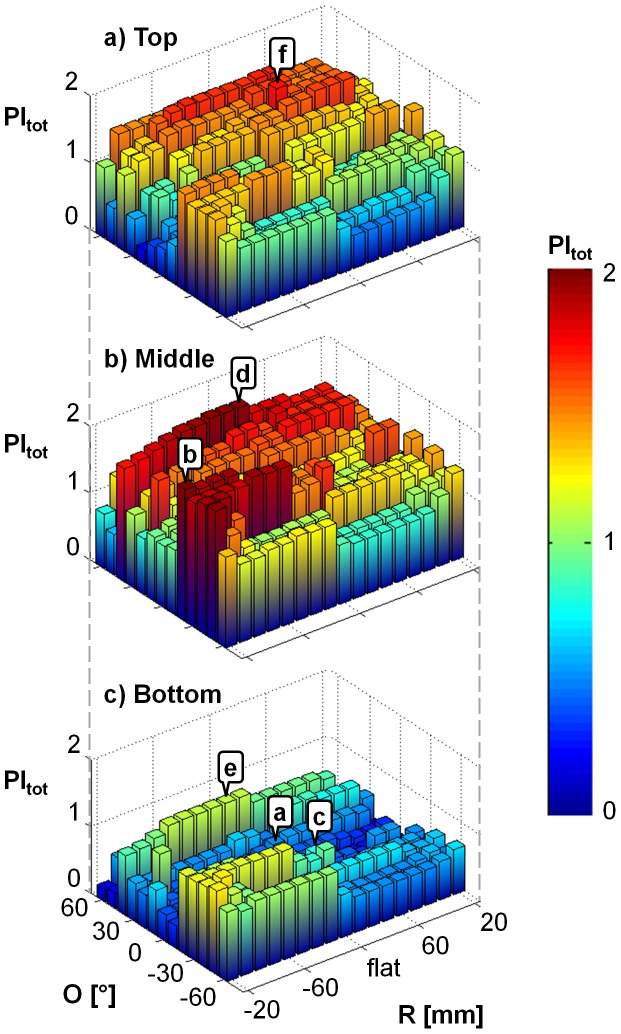
Total performance index results with respect to the different levels of keyboard radius of curvature (R) and keyboard orientation (O) for the (a) top, (b) middle and (c) bottom keyboard locations (L). Each bar on the graphs corresponds to the performance index for a different design. Data points identified by letters correspond to the highlighted designs presented in Figure 6 and discussed in the text.

We created 3D bar graphs to compare performance indices across keyboard designs with respect to each of the three design variables (Figure 5). From these graphs, we identified keyboard designs that represented local maxima and minima based on their total performance index, which indicated designs that performed well or poorly, respectively.

To determine the effect of each design variable on the predicted performance and since the data were not normally distributed, we used Kruskal-Wallis tests (significance level α = 0.05). For these analyses the dependent variable was the total performance index (PI_TOT_) (663 observations) and the independent variables were the design variables (radius of curvature (17 levels), orientation (13 levels), and location on the screen (3 levels)).

We accomplished all data processing and created bar graphs using Matlab (Version 2012b, The MathWorks, Natick, MA), and we carried out statistical analyses using SPSS Statistics for Windows, Version 20.0 (IBM Corp. , Armonk, NY).

## Results

**Figure 6:**
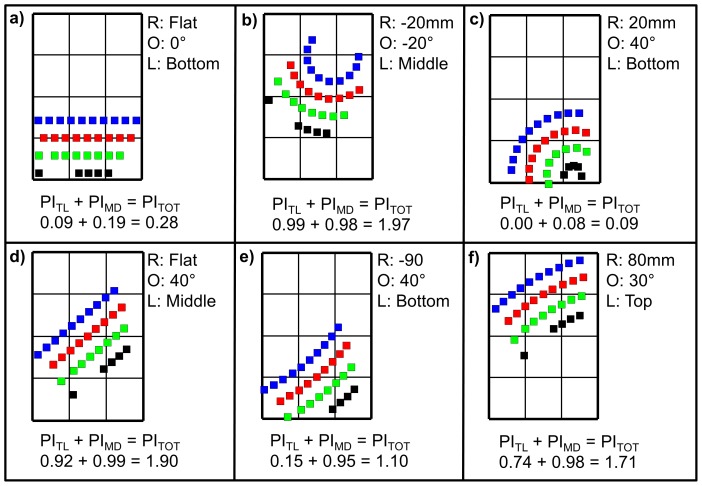
Highlighted designs with identification letter corresponding to the data points identified on Figure 5. Each design represents a specific combination of R (keyboard radius of curvature), O (keyboard orientation with respect to vertical), and L (keyboard vertical location on the screen). The performance index for tap location (PI_TL_), movement direction (PI_MD_), and the total performance index (PI_TOT_) are specified for each highlighted design. a) The standard keyboard; b) Best overall; c) Worst overall; d) Local maximum in the middle location; e) Local maximum in the bottom location; f) Best for the top location.

The current handheld device standard keyboard had a low performance index score compared to other designs. Its total performance index (PI_TOT_ = 0.28) ranked 638^th^ out of the 663 designs considered in this study. The design with the best total performance index was for R = −20 mm, O = −20°, L =  Middle with a PI_TOT_ of 1.97 (Figure 6b). Its tap location performance index (PI_TL_) and movement direction performance index (PI_MD_) ranked 7^th^ and 14^th^ out of the 663 designs considered, respectively, summing to the 1st ranked total performance index (PI _TOT_). The design that performed the worst was for R = 20 mm, O = 40°, L =  Bottom with a PI_TOT_ of 0.09 (Figure 6c).

Results from the Kruskal-Wallis tests suggest that keyboard location (L) had the greatest effect on the total performance index (H = 299.21, p<0.01), followed by keyboard orientation (O) (H = 107.02, p<0.01) (Table 2). Keyboard radius of curvature did not have a significant effect on the total performance index (H = 13.01, p = 0.67). Designs in the middle keyboard location were associated with the greatest total performance indices, followed by designs in the top and bottom locations (Figure 5c). From post-hoc analyses, PI_TOT_ variations across keyboard locations were mostly affected by PI_TL_, while variations of PI_TOT_ within keyboard locations were a function of both PI_TL_ and PI_MD_. Keyboard orientations that led to the greatest total performance indices overall were O = 40° and O = −30°.

Several local performance maxima and minima were apparent on the bar graphs (Figure 5). For the top keyboard location, performance increased with increasing O, with minima when O = 0° and negative R's. For the middle keyboard location, there were maxima when O = −30° and negative R's, and when O = 40° and negative R's. Additionally, there were minima when O = −10° and negative R's, and when O = −60° and positive R's. For the bottom keyboard location, PI_TOT_ increased as O decreased, and there were maxima when O = 40° and −40° for all negative R's, and minima for all O = 60°.

## Discussion

Our aim was to develop and demonstrate a handheld device keyboard design evaluation tool based on preexisting thumb motor performance data for different thumb movement directions and tap locations on a handheld device. The results support the hypothesis that the current standard keyboard for handheld devices performs poorly compared to other designs (Figure 6a). The keyboard's orientation and vertical location affected predicted user performance.

The use of pre-existing data allowed us to explore 663 different keyboard layouts providing direction for future testing. To test these different designs with human subjects would take a substantial amount of time. This study's strength is that it allows designers to narrow the design solution space by pre-selecting designs that optimize thumb motor performance, and understanding why motor performance is optimized, before moving on to user testing.

Previous studies have determined possible key locations that promote thumb performance, but this study is the first to propose data-driven QWERTY keyboard layout designs for single-handed use. Keys located in the middle and along the top-right/bottom-left diagonal of a handheld device screen and thumb movements along this orientation have been found to promote right thumb performance for single-handed use during tapping tasks [5], [8]–[9], [22]. These studies also suggest that keys located in the bottom right corner of the device should be avoided. The present paper supports these results, and utilizes basic data to propose, evaluate and compare actual QWERTY keyboard designs for the more complex task of typing.

Thumb performance was not equally sensitive to all design parameters. From the three design variables considered, results from the Kruskal-Wallis tests suggest that designers should first focus on the keyboard's vertical location on the screen as it contributed the most to the variation in total performance overall. The middle keyboard location was associated with the highest performances out of the three locations because tapping in the middle of the screen involves more neutral thumb postures compared to extended postures for designs in the top location, or flexed postures for designs in the bottom location [9]. The keyboard's orientation is the second most important design variable that designers should focus on. Orientations of O = 40° and O = −30° led to an increase in the frequency of thumb movement directions that promote thumb motor performance such as movements along the SW/NE orientation, and in the S to N direction. Designers can modify the keyboard's radius of curvature third, and this can be done by inspecting specific performance maxima and minima since not all regions of the parameter space are equally sensitive to curvature.

Our analysis revealed that several options may be available to increase thumb performance relative to the standard keyboard design (Figure 6a). The design with the greatest total performance index was design “b” (Figure 6b). However, the location of this design in the middle of the screen may impact other parameters such as usability. Additionally, the aesthetics of design “b” deviate substantially from the more familiar standard keyboard design. Design “d” was another local maximum for the middle location and therefore represents an alternative option that may be more familiar for users. Design “f” represented a local maximum in the top location because it involved more keys in the top right location, which was a location associated with the greatest thumb motor performance, and fewer in the top left compared to other designs in the top location. Moreover, design “f” placed the spacebar in tap location 6, which was associated with the second greatest thumb motor performance out of all the tap locations (Figure 2). Designs in the top location would involve placing the text below the keyboard, which represents an important usability shift from the current norm, which must be considered during the design process along with the factors investigated in this study (i.e., tap location and movement direction).

Minor alterations to the standard keyboard layout can potentially result in decreases in performance. For example, the design with the poorest total performance index was design “c”, which maintained the keyboard in the bottom location but rotated it relative to the thumb (Figure 6c). Although design “c” may appear more accommodating to the range of motion of the thumb, the spacebar was located entirely within the bottom right tap location. The spacebar is one of the most frequently tapped keys during typing, therefore design “c” involved several taps in tap location 12, which was the location associated with the poorest thumb motor performance because it requires an extreme thumb posture in flexion [9]. Design “c” also involved an increase in the frequency of thumb movements in the SE direction, which was associated with the poorest motor performance among all movement directions (Table 1). Changing the keyboard's radius of curvature to −90 mm such as for design “e” (Figure 6e) curved the spacebar such that it was not entirely in the bottom right corner tap location and extended the other keys, thus providing improved PI_TL_ and PI_MD_ over design “c” while keeping the same orientation as design “c” (i.e., 40°). Additionally, design “e” represented a substantial improvement on the standard keyboard design (i.e., Figure 6a) with respect to PI_MD_ (0.95 for design “e” compared to 0.19 for design “a”). The improvements in predicted performance associated with relatively minor changes to keyboard orientation demonstrate the sensitivity of performance and the potential importance of data-driven approaches to interface design.

Several limitations should be considered when interpreting these results. First, the evaluation tool did not consider other factors that are important in the design process such as aesthetics, learnability and where the text would be displayed. However, our results could contribute to a more comprehensive design process and do not prevent consideration of other factors. Next, a 375 character input text was used even though texts that are most frequently typed on handheld devices may be shorter such as status updates and “tweets” on social media applications. A 375 character text was chosen because it provided low variability across texts, and therefore provided a dependable parameter in the evaluation of each design. Additionally, the evaluation tool presented is focused on single-handed interaction and may not be generalizable to techniques that involve a two-handed grip such as thumb-typing on a tablet, or techniques that involve typing with the finger(s) instead of the thumb. Next, calculation of the total performance index as the sum of the movement direction and tap location indices in this study was based on an assumption that both factors contribute equally to the thumb's total motor performance, which may not be the case in practice.

Despite the limitations relating to the assumptions involved in developing a predictive model, we believe that this model presented here can be trusted as a useful tool for designers of handheld device keyboards because it is based on empirical data that utilizes scalable motor control paradigms, specifically Fitts' Law. The use of Fitts' law models in HCI has been prevalent since the 1950's to predict user performance for pointing tasks [20]. ISO standards have been developed based on this research [23], and an expansion of Fitts' paradigm has been applied, using a task analysis approach, to the development of predictive models for complex tasks such as typing on a keyboard [12] [14]–[17]. Additionally, applications of similar predictive models have been reported [24]–[25]. Despite their widespread use and applicability, predictive models such as the one presented here must be used with caution as they do not consider the extent of all factors that relate to the acceptance and usability of a new design. For example, as mentioned above, the proposed model does not consider aesthetics nor cognitive aspects related to the task (i.e., mental preparation required before accomplishing the different pointing tasks involved in typing). These omissions were deliberate to allow designers the freedom of choosing among designs that optimize ergonomics relating to biomechanical factors without any other constraints.

The design evaluation tool that we have developed has several potential applications. First, the tool can be used to generate hypotheses about the sensitivity of thumb motor performance to different design parameters. Second, the tool can provide a platform for the systematic inclusion of additional design parameters to make performance predictions. Finally, the tool provides the ability to compare a large number of designs without requiring prototypes nor users. However, prototype development and testing on actual users is required to determine if a particular design performs better than another in practice, and to validate our assumption, based on Fitts' Law, that performance data based on repetitive motions can be translated to the non-repetitive motion of typing.

Further development could involve including other design variables to the model such as visual access and postural measures. The evaluation tool described here indirectly considers postural measures since the performance indices used in the model have been found to be associated with thumb/wrist postures [9], but the direct inclusion of postural measures in the model may be used to develop a musculoskeletal injury risk model for handheld device use. Levels of thumb/wrist postures required to tap on different locations of the screen could be considered as the evaluation criteria instead of motor performance to identify designs requiring extreme postures that may lead to excessive musculoskeletal loading.

## Conclusions

Our results support the hypothesis that standard virtual keyboard layouts may not maximize thumb typing performance. Data-driven predictions of thumb performance as a function of keyboard location, orientation, and curvature suggest that several options exist to improve upon the standard handheld device keyboard design. Designs that resulted in the greatest predicted performances included keyboard designs in the middle location, designs that were oriented either at 40° or −30° with respect to vertical, and designs in which the spacebar was away from the bottom right corner of the device. Additionally, handheld device keyboard designers wanting to promote thumb use should consider modifying the keyboard location first, followed by its orientation and radius of curvature. The evaluation tool described in this study could be used to assist in the design process for evaluating keyboard designs in terms of their ability to promote thumb use.

## Supporting Information

Appendix S1 **Input Text.**
(DOCX)
