# Supplementary material for: A Data-Driven Design Evaluation Tool for Handheld Device Soft Keyboards
Source: PLoS One. 2014 Sep 11;9(9):e107070. doi: 10.1371/journal.pone.0107070 (PMC4161376; doi:10.1371/journal.pone.0107070)
Supplement: Appendix S1 — Input Text. (DOCX) [file pone.0107070.s001.docx]

“They gave up a quick 17 straight points. They even allowed the 49ers to get within 2 points. And, with less than 2 minutes to go, they were on the verge of losing the game. Unfortunately, this sequence - of positive momentum, a sudden negative shock, and a subsequent sapping of growth energy - has been a too-familiar one for the U.S. economy in recent years. Indeed, many economists feel that the U.S. still faces such a risk today. Gradual job creations”
